# Depressive symptoms in older adult couples: Associations with dyadic physical health, social engagement, and close friends

**DOI:** 10.3389/fpsyt.2022.989182

**Published:** 2022-09-13

**Authors:** Lyndsey M. Miller, Joel S. Steele, Chao-Yi Wu, Jeffrey Kaye, Hiroko H. Dodge, Mitzi M. Gonzales, Karen S. Lyons

**Affiliations:** ^1^School of Nursing, Oregon Health and Science University, Portland, OR, United States; ^2^Oregon Center for Aging and Technology, Oregon Health & Science University (OHSU), Portland, OR, United States; ^3^National Institute on Aging (NIA) - Layton Aging and Alzheimer's Disease Research Center, OHSU, Portland, OR, United States; ^4^Department of Psychology, Portland State University, Portland, OR, United States; ^5^Glenn Biggs Institute for Alzheimer's and Neurodegenerative Diseases, University of Texas Health Sciences Center San Antonio, San Antonio, TX, United States; ^6^William F. Connell School of Nursing, Boston College, Chestnut Hill, MA, United States

**Keywords:** social factors, depression, dyadic health, couple (spouses), gender

## Abstract

**Objective:**

The objective of this study was to examine associations between level of depressive symptoms in older adult spouse/partner couples and their physical health and social factors (social activity and number of close friends).

**Methods:**

Using data from 116 community-dwelling couples (age 76.2 ± 8.5), we simultaneously analyzed associations between depressive symptoms (Geriatric Depression Scale, range 0–11) and dyadic physical health, engagement in social activities, and connectedness with close friends.

**Results:**

Greater engagement in social activities was associated with fewer depressive symptoms in men, whereas more close friendships were associated with fewer depressive symptoms in women, controlling for partner effects, age, education, and cognitive function, with good model fit. Additionally, more disparate physical health within the couple (latent incongruence score) was associated with greater depressive symptoms in men.

**Discussion:**

Less social activity and fewer close friends were associated with depressive symptoms in older adult couples, but may be distinctly influential depending on gender and in the context of the older adult couple's physical health.

## Introduction

It is well-known that major depressive disorder is less common among adults ages 65 and older than in adults of any other age in the United States (1, 2). Yet, the prevalence of mild depressive symptoms (i.e., subsyndromal depression) is similar across all community-dwelling adult age groups in the United States (roughly 10%), and the consequences of subsyndromal depression for older adults in particular include elevated risk of morbidity and lower quality of life (3–5). Known protective factors against depressive symptoms among older adults include better physical health (6), and social factors such as social engagement (7–9) and social connectedness (10–12). While the majority of older adults are married or have a co-habiting intimate partner relationship (13), few studies have considered the context of the physical health of the couple (rather than the individual), or the added and potentially distinct benefits beyond marriage of engaging in social activity and maintaining social connectedness with friends.

### Dyadic theory and context for mental health in couples

Research provides consistent evidence of the health benefits of being married or partnered (14, 15), yet there is also clear indication that older adults' health problems can negatively impact their partners' mental health (16–19). Incongruent physical health in a dyad (i.e., differences between partners' health) has implications for imbalances in the relationship, shifting social roles (e.g., caregiving), and overall mental health (20). Thus, it is important to look beyond individual-level physical health and consider how the dyad's health as a unit may be contributing to depressive symptoms.

**Interdependence theory** and related research explains that for older adults who are married or partnered, their health, behaviors and social milieu are also interdependent and connected as a couple (10, 21, 22). There are gender differences, though, in the manner and degree to which one construes their identity as interdependent (23). Markus and Kitayama (24) posit the interdependent self-construal is more magnified in women, who are socialized to be caring and closely bonded with others; whereas, the independent self-construal is magnified in men, who are more socialized to stand out from their peers and lead. Alternatively, a more relational typology of interdependence is proposed as dominant in females (i.e., interdependence with one's partner and other dyadic bonds such as close friends), vs. a more collectivist typology of interdependence in males (i.e., self-construed as is interdependent with the group or community to which one belongs) (25).

Spouses and close intimate partners are vital social ties that contribute to wellbeing and also enhance social opportunities (26). Yet little dyadic research has examined the distinct roles of other social factors–beyond the effects of one's partner–on the mental health of the older couple. Social-Emotional Selectivity Theory, though not a dyadic theory, posits that older adults experience an intrinsic motivational shift to de-emphasize potentially negative social and emotional influences and maximize positive ones in accordance with the degree to which it is perceived that there is limited time left to achieve one's life goals (27). The ability to identify and select positive social and emotional influences and give less attention to negative ones contributes to enhanced emotional regulation and helps explain the paradox of lower rates of depression among older adults despite greater physical health challenges (28). By selecting the marital context for examining the influence of social factors on depression, it allows us to examine the additive benefit of social connectedness and social activity engagement beyond the support and enrichment that is available through one's own spouse. Additionally, it allows us to examine partner effects that may be present due to the “marital capital” that is gained by having access to a partner's social network and activities (10, 29).

### The protective role of social factors

Social connectedness with close friends and engaging in social activities are clearly related (30), but the two constructs and the benefits they convey are also be distinct. Close friends are the strongest forms of social connections and are considered vital relationships: they provide social support, and prevent social isolation, loneliness (30–33). More frequent contact with close friends is also associated with fewer depressive symptoms in older adults (11) and among other age groups (34). Social activities, on the other hand, can be engaged in without close friends (e.g., playing a game with an acquaintance), but enrich and involve older adults in a facet of life that is beyond one's necessary activities of daily living (35). Social activities are also associated with a lower risk of developing depressive symptoms as well as an improvement in depressive symptoms when present (8, 9).

It is possible to have and benefit from close friends without engaging in social activities, just as it is possible to engage in and benefit from social activities without having close friends. Though the two can also be interrelated, it is necessary to simultaneously examine the distinct protective benefit that each concept may offer against depressive symptoms. In a rare example, one study of older adults examined the impact of diverse social ties and daily activities on behaviors and daily mood, and found that interactions with close friends and engagement in diverse behaviors (i.e., social activities) both improved daily positive mood, but did not decrease daily negative mood (36). Although this study provided important insight into the minutia of social factors and daily mood shifts, it is still unclear what the distinct protective influences of social connectedness and engagement in social activities are against depression in older adults. Further research is needed in order to understand and help promote the benefits of social factors for mental health.

### Study objective

The overall objective of this study was to examine the associations between dyadic health, social activity and social connectedness on depressive symptoms in older adult couples. We expected to observe: (1) associations between one's own social activities and close friends on one's own level of depressive symptoms, such that higher levels of both social measures would be associated with lower levels of depression; (2) one's partner's levels of social measures would also be related to depressive symptoms in couples; and (3) an association between incongruent dyadic physical health and depressive symptoms.

## Methods

### Sample

Cross-sectional data from community-dwelling older adult couples in the Pacific Northwest region of the United States (primarily Portland, Oregon and surrounding areas) who participated in observational studies between the years of 2011–2019 were included in this secondary analysis. Each of the three original studies' recruitment and inclusion criteria are detailed in previously published papers (37–39). The inclusion criteria for minimum age of the primary participant enrolling: 50 years of age or older (39), 62 years of age or older (37), and 80 years or older (38), respectively, however there was no age restriction on co-habiting spouses/partners for the original studies. For this analysis data from participants who lived alone were excluded, and data from all participants who were living with a spouse/partner at the time of completing the baseline measure of depressive symptoms were included in this analysis. In total, the current study included data from 116 co-habiting spouses or partners (a total of 232 older adults). All data were collected prior to the start of social distancing and other social restrictions imposed during the COVID-19 pandemic. A clinical assessor from the study team collected data from participants in-person on health variables (depressive symptoms, physical health, and cognitive function). All other study variables were collected with an online survey (*via* Qualtrics) at the same time point as the in-person visit. Ethical approval was obtained from a university health center IRB for data to be stored and shared through a data repository, which was accessed for the current study.

### Measures

#### Depressive symptoms

Depressive symptoms were measured with the 15-item Geriatric Depression Scale (GDS), which is a valid and reliable self-report screening instrument for assessing depressive symptoms in older adults (40). A research clinician completed the GDS with participants during in-person study visits. Responses are yes/no and the scale range is 0–15, with 1 point assigned for each item endorsed with a yes response. Depressive symptoms were treated as a continuous variable in this study, however the clinical diagnostic relevance of scores of 0–2 can be interpreted as no depression, scores of 3–5 as subsyndromal depression, and scores of 6+ as syndromal depression (3).

#### Engagement in social activity

Engagement in social activity was measured by the frequency (0 = *rarely or never*, 1 = *yearly*, 2 = *monthly*, 3 = *weekly*, 4 = *daily*) of engaging in eight activities: visits from others, visits to others, eating meals out in restaurants, spending time doing hobbies or games, attending clubs or group meetings, attending a class, attending church or religious services, and travel out of town. Items were averaged across all eight activities for a scale score range of 0–4. This measure was adapted from the Brief Assessment of Social Engagement scale (41).

#### Social connectedness

Social connectedness from close friends was self-reported with a single item on a scale of 0–5, where a response choice of 5 = 9 or more close friends, a score of 4 = 5–8 close friends, a score of 3 = 3 or 4 close friends, a score of 2 = 2 close friends, 1 = 1 close friend, and 0 = 0 close friends.

#### Physical health

Physical health was measured using the modified Cumulative Illness Rating Scale (M-CIRS), which has 14 items, each representing the presence of an illness type that is rated on a 5-point Likert-type scale of severity (0 = “None” to 4 = “Extremely Severe”) (42). The total score ranges from 0 to 56, with a higher score indicating more severe illness, or less physical health. A research clinician completed the M-CIRS with participants during in-person study visits. Reliability of the M-CIRS was not calculated for this study due to the nature of the scale's items, which each focus on a distinct source of pathology (e.g., cardiovascular or psychiatric). Thus, we would not necessarily expect items to be correlated. Previous research has established the test-retest reliability of the CIRS-G and its validity with a sample of community-dwelling older adults (42).

#### Cognitive function

Cognitive function was measured using the 30-point Mini-Mental State Examination (MMSE) (43) among 68 couples, and the MMSE conversion equivalency score (44, 45) was used for this analysis for the remaining 41 couples who completed the 30-point Montreal Cognitive Assessment (MoCA-30) (46). The MMSE is designed for clinician assessment of 11 cognitive domains spanning aspects of orientation, working memory, language, delayed recall, attention, and comprehension. The scale range is 0–30, with higher scores indicating higher cognitive function. The resulting variable for cognitive function in this sample was a unified score from the MMSE/MMSE conversion scale. Although there are differences in the emphasis on domains measured by the MoCA vs. the MMSE, both scales are widely used and it is often necessary to convert scores from one scale to the other (44, 45).

#### Demographic variables

Demographic variables included self-reported gender identity, age in years, race, ethnicity, and education in number of years completed. Age and education were included as covariates based upon previous literature indicating the effects of education on social factors and depressive symptoms (7, 9, 10, 12, 36), as well as our theoretical framework specifying that adults become increasingly more selective of social influences as they age (27), and that education expands the intrinsic and extrinsic resources available to older adults' for emotional regulation (28).

### Analytic approach

#### Dyadic modeling

In order to examine the relationship between social factors and depressive symptoms in older adult couples, we adopted a dyadic modeling perspective wherein measures and outcomes for both partners are modeled simultaneously. Specifically, the Actor Partner Interdependence Model (APIM) (47) was used to assess both individual level, or actor effects, along with any potential reciprocal, or partner, effects of social factors, physical health, and cognitive function on levels of mental health. Structural equation modeling (SEM) was used since it provides a straightforward way to specify individual and reciprocal effects for both partners simultaneously. This approach also addresses the dependence inherent in measuring partners in a relationship, wherein individual socializing or health behaviors are interdependent. Such interdependence violates traditional statistical assumptions which treat each individual and their observations as independent.

**Figure 1:**
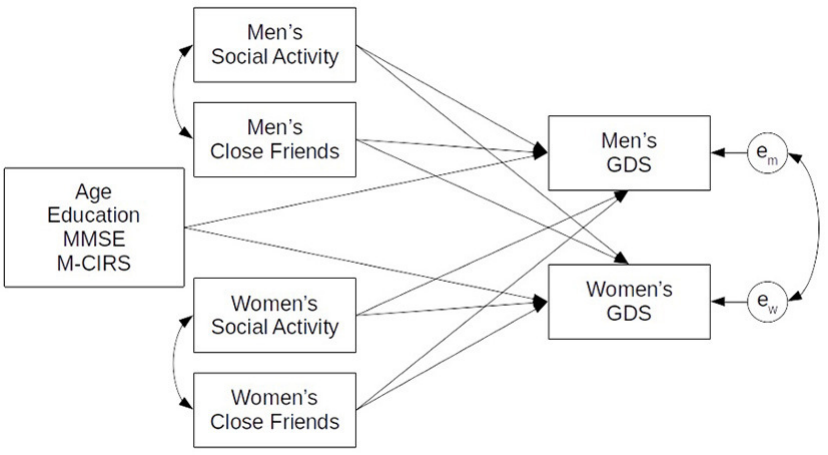
APIM model of social factors and physical health on depressive symptoms.

The integrated model examined the associations between social factors, measured through close friends (*friends*) and social activities (*soc8*), on depressive symptoms (*GDS*) of individuals living together in a couple. In the model, levels of *GDS* for both partners were associated by individual levels of *soc8* and *friends* as well as *soc8* and *friends* from their partner. A structural model diagram of this APIM model is illustrated in Figure 1. This allowed us to assess both types of effects, actor and partner, simultaneously while also addressing missing data through use of full information maximum likelihood (FIML) for model estimation. All models were estimated using R version 3.6.3 (2020-02-29), and the package lavaan 0.6-5 for structural equation modeling. Syntax for these analyses is provided in Appendix A.

Model fit was assessed using a combination of indices including the Chi square (*p*-values > 0.05 indicative of close model fit), the comparative fit index (CFI) and the Tucker Lewis Index (TLI), both of which compare the fit of the specified model to that of a null model (values > 0.95 for both indices indicative of good model fit), the Root Mean Square Error of Approximation (RMSEA), a per degree of freedom index of model fit (values < 0.05 indicative of good model fit), and the Standardized Root Mean Square Residual (SRMR) with values < 0.08 considered a good fit (48–50). Significance is reported at the level of *p* < 0.05.

#### Procedure for analyzing dyadic physical health

In order to incorporate physical health from a dyadic health perspective (51) in our analyses, we adopted a second-order approach wherein individual scores on a measure are replaced by a dyad level average and a dyad level difference (incongruence) score (52, 53). Specifically, we incorporated two latent variables (procedure specified in R using the code in Appendix A). The first indexed the average level of physical health by constraining the loadings from the latent variable to both partner measures to be equal to one. The other latent variable coded the incongruence between partners' physical health by setting one partner's loading to 0.5 and the other to −0.5 thus indexing the difference between partner measures. With these two latent measures specified, we were able to assess the associations between both the couple level average and the degree of incongruence in physical health on each partner's level of depressive symptoms.

## Results

Depressive symptoms were low on average for both women (1.33 ± 1.68) and men (1.92 ± 2.62), but among the 116 couples, scores for 14 women (12%) and 19 men (16%) were indicative of subsyndromal depression, and scores for 4 women (3%) and 9 men (8%) were indicative of syndromal depression. Women and men in the sample were, on average, 75 and 78 years of age, respectively, of predominantly non-Hispanic white ethnicity/race (91%), and had obtained on average some college education. Means and standard deviations for all study variables are provided in Table 1. Pairwise correlations between study variables are provided in Table 2.

**Table 1:** Descriptive statistics.

	***n***	**Mean**	***SD***	**Min**	**Max**	**Skew**
**Male**						
Depressive symptoms	115	1.92	2.26	0.00	11.00	1.83
Cognitive function	111	26.32	3.87	8.00	30.00	−2.18
Physical health	109	22.83	3.64	15.00	34.00	0.14
Social connectedness: friends	103	2.95	1.58	0.00	5.00	−0.32
Social activity engagement	112	1.83	0.66	0.38	3.43	−0.10
Age in years	116	77.85	8.16	61.00	100.50	0.11
Education in years	116	15.96	2.78	8.00	20.00	−0.39
**Female**						
Depressive symptoms	116	1.33	1.68	0.00	10.00	2.08
Cognitive function	112	28.20	2.23	15.00	30.00	−2.60
Physical health	96	21.27	3.64	15.00	36.00	1.25
Social connectedness: friends	103	3.29	1.28	0.00	5.00	−0.49
Social activity engagement	108	2.02	0.53	0.38	3.17	−0.53
Age in years	116	74.51	8.51	56.90	89.90	0.10
Education in years	116	15.20	2.31	10.00	20.00	−0.13

**Table 2:** Correlations between study variables using pairwise completion.

	**1.**	**2.**	**3.**	**4.**	**5.**	**6.**	**7.**	**8.**	**9.**	**10.**	**11.**	**12.**	**13.**	**14.**
1. GDS.m	–													
2. mmse.m	−0.188	–												
3. mcirs.m	0.216	−0.092	–											
4. friends.m	−0.036	0.135	0.038	–										
5. soc8.m	–**0.301**	0.171	−0.157	**0.405**	–									
6. age.m	−0.014	−0.128	0.026	0.197	0.037	–								
7. educ.m	−0.010	0.171	−0.082	0.074	0.187	0.153	–							
8. GDS.f	0.167	0.001	0.073	−0.049	−0.195	−0.125	−0.060	–						
9. mmse.f	0.045	−0.209	−0.114	0.061	0.117	0.101	0.211	−0.239	–					
10. mcirs.f	−0.128	−0.163	0.075	−0.041	−0.136	0.059	−0.262	**0.284**	−0.149	–				
11. friends.f	−0.035	−0.043	−0.047	0.221	0.046	0.148	0.145	–**0.347**	0.230	−0.115	–			
12. soc8.f	−0.161	−0.080	−0.084	0.178	**0.466**	0.011	0.155	–**0.333**	**0.300**	−0.212	**0.356**	–		
13. age.f	−0.069	0.035	−0.065	0.194	0.058	**0.815**	**0.281**	−0.043	−0.011	0.074	0.084	−0.019	–	
14. educ.f	0.013	−0.185	−0.158	0.062	0.198	0.115	**0.400**	−0.084	0.189	−0.169	0.191	0.261	0.208	–

Bold values indicate the *p*-value is significant at the level of < 0.05. GDS stands for Geriatric Depression Scale.

Our dyadic model included depressive symptoms for both partners which were simultaneously regressed on social measures (activity engagement and number of close friends) from both the individual and their partner, and dyadic health, controlling for age, cognitive function, and years of education (Table 3). Greater engagement in social activities was associated with fewer depressive symptoms in men, whereas more close friendships were associated with fewer depressive symptoms in women, controlling for partner effects (NS), age (NS), cognitive function (NS), and education (NS), with good model fit.

**Table 3:** Associations between social factors and depressive symptoms within the context dyadic physical health.

	**Est**	**S.E.**	***p*-value**	**CI lower**	**CI upper**	**Std.all**
**GDS male regression**						
Social connectedness: friends_m_	0.22	0.15	0.15	−0.08	0.52	0.15
Social connectedness: friends_f_	−0.05	0.18	0.77	−0.42	0.31	−0.03
Social activity engagement_m_	–**1.00**	**0.39**	**0.01**	–**1.77**	–**0.22**	–**0.29**
Social activity engagement_f_	−0.31	0.49	0.52	−1.27	0.65	−0.07
Dyadic physical incongruence	**0.13**	**0.05**	**0.01**	**0.04**	**0.22**	**0.26**
Dyadic physical health average	−0.01	0.09	0.87	−0.18	0.16	−0.02
Cognitive function_m_	−0.12	0.06	0.06	−0.25	0.01	−0.21
Cognitive function_f_	0.05	0.10	0.58	−0.13	0.24	0.05
Age in years_m_	−0.02	0.03	0.47	−0.07	0.03	−0.07
Education in years_m_	0.04	0.08	0.62	−0.11	0.19	0.05
**GDS female regression**						
Social connectedness: friends_f_	–**0.32**	**0.13**	**0.02**	–**0.58**	–**0.06**	–**0.25**
Social connectedness: friends_m_	0.14	0.11	0.21	−0.08	0.35	0.13
Social activity engagement_f_	−0.62	0.36	0.08	−1.32	0.08	−0.20
Social activity engagement_m_	−0.30	0.29	0.30	−0.86	0.27	−0.12
Dyadic physical incongruence	−0.05	0.03	0.12	−0.11	0.01	−0.14
Dyadic physical health average	0.09	0.06	0.13	−0.03	0.22	0.15
Cognitive function_f_	−0.09	0.07	0.19	−0.22	0.04	−0.12
Cognitive function_m_	−0.01	0.04	0.90	−0.08	0.07	−0.01
Age in years_f_	−0.01	0.02	0.44	−0.05	0.02	−0.07
Education in years_f_	0.06	0.07	0.37	−0.07	0.19	0.08
**Correlated residuals**						
GDS_m_ GDS_f_	0.46	0.28	0.11	−0.10	1.02	0.16
**Variances**						
GDS_m_ GDS_m_	**4.04**	**0.55**	**0.00**	**2.97**	**5.12**	**0.79**
GDS_f_ GDS_f_	**2.11**	**0.29**	**0.00**	**1.55**	**2.68**	**0.77**
**Latent incongruence intercept**						
Dyadic physical health average	**22.05**	**0.26**	**0.00**	**21.54**	**22.56**	**8.58**
Dyadic physical health incongruent	**1.51**	**0.49**	**0.00**	**0.55**	**2.46**	**0.32**

Model fit: $\chi_{\left(24\right)}^{2}$: 28.36, p = 0.25.

CFI: 0.89, TLI: 0.79.

RMSEA: 0.04 CI (0.00, 0.09).

SRMR: 0.05.

Bold values indicate the *p*-value is significant at the level of < 0.05. GDS stands for Geriatric Depression Scale.

Our model also simultaneously incorporated dyadic physical health, which allowed us to compare whether it was the overall level of dyadic health in the couple that was the salient factor for higher levels of depressive symptoms, or whether it was the incongruence/discordance between partners regarding physical health that was significant, since both of these aspects of dyadic physical health have potential implications for mental health (54). From the results in Table 3, it can be seen that greater incongruence in couples' physical health was significantly associated with males' depressive symptoms. The coding of the incongruence was such that positive values indicated higher levels of physical illness for males than for females. Thus, men exhibited greater depressive symptoms in this study when there was more incongruence between their own amount of physical illness and their (more often healthier) female partner's.

## Discussion

This study found that social activity and connectedness are positively associated with mental health in older adult couples, but appear to be distinctly influential depending on the individual's gender and in the context of the dyad's health. The findings of this study support our hypotheses that social factors may protect against depressive symptoms, however we did not find partner effects that suggested that social engagement or connectedness of one's partner influenced older adults depressive symptoms. This study builds upon the existing literature by highlighting the differential benefits of two distinct social factors–social activity engagement for men and social connectedness from close friends for women. This study also highlights the association between the couple's physical health and depressive symptoms, with a novel focus and methodological approach to dyadic incongruence in physical health.

A recent review of friendship in late life specifically noted the need for greater understanding of gender effects (30). In this study, our expectation for the first study hypothesis was supported, and we found that more close friends were associated with fewer depressive symptoms, however the finding was only among women in older adult couples, and not in men. This confirms findings from other recent studies of depressive symptoms in older adult couples (10, 29), and across a larger (non-dyadic) study that found women, but not men, report greater depressive symptoms when they lack a close friend (55). These findings also support theoretical explanations of gender differences in interdependence, and the importance that women place on social relationships (24, 25).

Although it is thus not a surprise that women significantly benefitted from more close friends, it was surprising that our expectation for the second study hypothesis was not supported and there was no partner/cross-over benefit to men. Husbands are often part of their wife's social network and thus may benefit from the marital capital (10). Our study adds to this body of literature by including the effects of engagement in social activities in the analysis. Indeed, another gender-specific finding of this study is that higher engagement in social activities were associated with fewer depressive symptoms in men, but not women. In the context of protecting against depressive symptoms, this distinct social influence may reflect “his and hers interdependence”, with a more collectivist typology related to shared social activities in men (25). Extending this to the marital context, these two distinct findings favor a gender-as-relational model from within a social construct (marriage) that is inextricably linked to health (56).

The results of this study also indicated support for our third study hypothesis with a negative association between incongruent dyadic physical health within couples on the mental health of men. This incongruence represents an imbalance in health within the couple that may disturb the homeostasis of the relationship, placing the couple in a vulnerable context where roles may shift (e.g., one partner providing care to another, or recognition that one partner is becoming more frail) (20, 57). Men in particular may feel less prepared or socialized for viewing themselves as being more frail or needing more care than their female partners. Recent research by Polenick et al. (54) found incongruence in type of chronic health conditions was significantly associated with higher depressive symptoms for husbands but not wives. An imbalance in physical health within couples may diminish the ability to remain as socially active and require renegotiations within the dyad regarding household tasks, lifestyle, and in some cases daily management of illness (e.g., diabetes). Although an emerging area of research, the research on dyadic health emphasizes the importance of understanding the implications of imbalance in health within dyads (and particularly couples) through the use of dyadic approaches and second-order dyadic variables (20, 53). These approaches are also crucial to fully evaluate interventions that are efficacious for the dyad, not just the individuals within it (20, 57).

### Limitations

We were unable to examine the influence of spousal relationship quality in this secondary dyadic analysis, which may help to further distinguish the benefits of social activities or social connectedness to older adults' mental health (11), or the potential interactions between types of social engagement and being in a spousal relationship with mental health in couples (58). It is possible that support from spouses would also mitigate the effect of incongruent dyadic health on depressive symptoms (59). Our definition of social connectedness from close friends is narrower than one that includes all types of social connections, and our measure is a single item. Further research is needed to understand if this study's findings translate to weaker social ties, and whether it holds with a more robust measure of social connectedness that takes into account dimensions of closeness such as having a confidant. We were also unable to examine the effects of social influences on depressive symptoms across non-binary gender identities or in same-sex couples in this study. It will be important to include same-sex couples in future studies to understand the unique context of this type of partnership, but also to help determine whether the gender differences found in different-sex couples hold true or if the finding is more reflective of the social role adopted in the relationship (i.e., gender as a social construct).

The average endorsement of depressive symptoms across men and women in our sample was low. It is possible that the strength of the association with social activities and/or connectedness with close friends may vary among individuals with greater depressive symptom severity, as well as couples from diverse cultures, backgrounds, and environments. Future research is needed to further explore the replication of these associations across samples. Finally, the data used for these analyses are cross sectional and therefore estimated effects are not causal. While social activity and connectedness may offer psychological benefits, having depressive symptoms may also reduce social activity and connectedness. However, given the alignment of these findings with prior research and theory, we feel confident that the estimated effects that resulted from our models will replicate in future studies. Ideally, longitudinal studies will examine our results to more definitively determine the direction of causality.

### Strengths and conclusions

This study adds to the literature on the importance of social activity engagement and social connectedness to depressive symptoms among older adults by examining these two distinct social factors simultaneously. This study also extends the findings to the vital context of the marital environment, which previous research has emphasized as necessary to consider when evaluating and treating depression in older adults (17). Lastly, this study takes into account the varying degrees of physical health within and across community-dwelling couples. There are clearly positive implications for the mental health of couples who are able to remain socially active and connected with close friends, and who remain physically healthy together.

## Data availability statement

Publicly available datasets were analyzed in this study. This data can be found here: https://octri.ohsu.edu/redcap/surveys/?s=EW4HKW47XH.

## Ethics statement

The studies involving human participants were reviewed and approved by Oregon Health and Science University IRB. The patients/participants provided their written informed consent to participate in this study.

## Author contributions

LM was primarily responsible for the manuscript writing and performed initial data analysis. JS created R code for the incongruence analysis. LM, JS, C-YW, MG, and KL contributed to the study design and manuscript writing. HD and JK were responsible for data collection, study supervision, and manuscript revisions. All authors contributed to the article and approved the submitted version.

## Funding

This work was supported in part by the National Institutes of Health (K01AG059839, P30AG008017, P30AG066518, P30AG024978, R01AG024059, and U2CAG0543701); the Merck Investigator Study Program (55172); and the Veterans Administration (IIR 17-144). The content is solely the responsibility of the authors and does not necessarily represent the official views of the National Institutes of Health or other funders.

## Conflict of interest

The authors declare that the research was conducted in the absence of any commercial or financial relationships that could be construed as a potential conflict of interest.

## Publisher's note

All claims expressed in this article are solely those of the authors and do not necessarily represent those of their affiliated organizations, or those of the publisher, the editors and the reviewers. Any product that may be evaluated in this article, or claim that may be made by its manufacturer, is not guaranteed or endorsed by the publisher.
