# Supplementary material for: Depressive symptoms in older adult couples: Associations with dyadic physical health, social engagement, and close friends
Source: Front Psychiatry. 2022 Sep 13;13:989182. doi: 10.3389/fpsyt.2022.989182 (PMC9513127; doi:10.3389/fpsyt.2022.989182)
Supplement: Supplementary file 1 [file Table_1.DOCX]

Appendix A. R syntax

## load the data

library(readxl)

socl_dat = read_excel("ORCATECH 116 dyads social.xlsx", na=".")

inp_cols = c("homeid","subid","age","educ","GDS","mmse","CDR","friends",

"soc_visit","soc_visitor","soc_meals","soc_class","soc_club",

"soc_church","soc_travel","soc_hobby","mcirs","soc8")

# compute social mean score

socl_dat$soc8 = apply(socl_dat[,grep("soc_",names(socl_dat))],1,mean,na.rm=T)

# subset per partner

socl_dat.M = socl_dat[socl_dat$Mdummy==1,inp_cols]

socl_dat.F = socl_dat[socl_dat$Fdummy==1,inp_cols]

# combine into wide format

socl_wide = merge(socl_dat.M, socl_dat.F, by=c("homeid"),

suffixes = c('.m','.f'))

# clean up

rm(list=c('socl_dat','socl_dat.M','socl_dat.F'))

# model columns, variables to be used in these analyses

mdl_cols = c('GDS.m','mmse.m','mcirs.m','friends.m','soc8.m','age.m','educ.m',

'GDS.f','mmse.f','mcirs.f','friends.f','soc8.f','age.f','educ.f')

# Table 1. descriptive statistics

dst = psych::describe(socl_wide[,mdl_cols])[,-c(1,5:7,10,12:13)]

# Correlations

ct = psych::corr.test(socl_wide[,mdl_cols],use='pairwise')

# SEM modeling

library(lavaan)

# Table 2. model

social_model = '

GDS.m ~ friends.m + friends.f +

soc8.m + soc8.f +

age.m + educ.m

GDS.f ~ friends.f + friends.m +

soc8.f + soc8.m +

age.f + educ.f

# covariances

GDS.m ~~ GDS.f'

social_model_fit = sem(social_model, data=socl_wide, missing='fiml', fixed.x=F)

# Table 3. model

mcirs_model = '

# include mcirs and predict GDS from this.

GDS.m ~ friends.m + friends.f +

soc8.m + soc8.f +

mcirs.m + mcirs.f +

mmse.m + mmse.f +

age.m + educ.m

GDS.f ~ friends.f + friends.m +

soc8.f + soc8.m +

mcirs.m + mcirs.f +

mmse.f + mmse.m +

age.f + educ.f

# covariances

GDS.m ~~ GDS.f'

mcirs_model_fit = sem(mcirs_model, data=socl_wide, missing='fiml', fixed.x=F)

# Table 4. model

incongruence_model = '

# define latent incongruence for mcirs

mcirs.int =~ 1*mcirs.m + 1*mcirs.f

mcirs.inc =~ .5*mcirs.m + -.5*mcirs.f

mcirs.m ~ 0

mcirs.f ~ 0

mcirs.m ~~ 1*mcirs.m

mcirs.f ~~ 1*mcirs.f

# latent variable intercepts

mcirs.int ~ 1

mcirs.inc ~ 1

# model

GDS.m ~ friends.m + friends.f +

soc8.m + soc8.f +

mcirs.inc + mcirs.int +

mmse.m + mmse.f +

age.m + educ.m

GDS.f ~ friends.f + friends.m +

soc8.f + soc8.m +

mcirs.inc + mcirs.int +

mmse.f + mmse.m +

age.f + educ.f

# covariances

GDS.m ~~ GDS.f

mcirs.int ~~ mcirs.inc'

incongruence_model_fit = sem(incongruence_model, data=socl_wide,

missing='fiml', fixed.x=F)
